# Loss of Satb2 in the Cortex and Hippocampus Leads to Abnormal Behaviors in Mice

**DOI:** 10.3389/fnmol.2019.00033

**Published:** 2019-02-12

**Authors:** Qiong Zhang, Ying Huang, Lei Zhang, Yu-Qiang Ding, Ning-Ning Song

**Affiliations:** ^1^Key Laboratory of Arrhythmias, Ministry of Education of China, East Hospital, Tongji University School of Medicine, Shanghai, China; ^2^Department of Anatomy and Neurobiology, Tongji University School of Medicine, Shanghai, China; ^3^State Key Laboratory of Medical Neurobiology and MOE Frontiers Center for Brain Science, Institutes of Brain Science, Fudan University, Shanghai, China; ^4^Department of Laboratory Animal Science, Fudan University, Shanghai, China

**Keywords:** Satb2, cerebral cortex, hippocampus, Satb2-associated syndrome, mouse behavior

## Abstract

Satb2-associated syndrome (SAS) is a genetic disorder that results from the deletion or mutation of one allele within the Satb2 locus. Patients with SAS show behavioral abnormalities, including developmental delay/intellectual disability, hyperactivity, and symptoms of autism. To address the role of Satb2 in SAS-related behaviors and generate an SAS mouse model, Satb2 was deleted in the cortex and hippocampus of Emx1-Cre; Satb2^flox/flox^ [Satb2 conditional knockout (CKO)] mice. Satb2 CKO mice showed hyperactivity, increased impulsivity, abnormal social novelty, and impaired spatial learning and memory. Furthermore, we also found that the development of neurons in cortical layer IV was defective in Satb2 CKO mice, as shown by the loss of layer-specific gene expression and abnormal thalamocortical projections. In summary, the abnormal behaviors revealed in Satb2 CKO mice may reflect the SAS symptoms associated with Satb2 mutation in human patients, possibly due to defective development of cortical neurons in multiple layers including alterations of their inputs/outputs.

## Introduction

Special AT-rich sequence-binding protein 2 (Satb2) is a transcription factor that regulates chromatin remodeling and gene expression via interactions with genomic nuclear matrix attachment regions, and it plays a pivotal role in the development of multiple organs. In skeletogenesis, Satb2 is essential for the craniofacial patterning and bone formation (Dobreva et al., 2006). In brain morphogenesis, Satb2 is required for the development of both callosal and subcortical projection neurons in the neocortex (Alcamo et al., 2008; Britanova et al., 2008; Leone et al., 2015; McKenna et al., 2015). In Satb2 conventional knockout mice and Satb2 CKO mice, most of callosal neurons do not send axons to the contralateral cortex (Alcamo et al., 2008; Leone et al., 2015). Recently, it has been reported that Satb2 is also required for the differentiation of a subset of spinal interneurons (Hilde et al., 2016).

In humans, the deletion or mutation of one allele within the Satb2 locus results in a disorder called Satb2-associated syndrome (SAS). Patients with SAS show craniofacial anomia, growth retardation, and behavioral abnormalities such as developmental delay/intellectual disability, hyperactivity, and symptoms of autism (Usui et al., 2013; Zarate and Fish, 2017; Zarate et al., 2018). In addition, about 29% of patients with SAS show abnormal white matter and about 8% have a small corpus callosum, as revealed by brain imaging (Zarate et al., 2018), which may contribute to the behavioral abnormalities.

Available mouse models with a selective deletion of Satb2 in different neuronal types and brain regions have allowed researchers to explore the neurobiological basis of SAS in humans. Defective social, fear, and spatial memory have been reported in Satb2 conditional knockout (CKO) mice with a deletion of Satb2 in CamKII-Cre-expressing neurons and heterozygous Satb2 mice (Jaitner et al., 2016; Li et al., 2017). Unlike Satb2 KO mice, which die after birth with multiple defects (Dobreva et al., 2006), CKO mice can survive for at least 1 year and have normal gross appearance.

To better understand SAS, particularly its autistic and behavioral symptoms, we generated Satb2 CKO mice in which Satb2 was deleted in the cerebral cortex and hippocampus, which are two major brain regions with high levels of Satb2 expression (Huang et al., 2013a) and are likely to be involved in the SAS-related behavioral phenotypes. Here, we reported that the deletion of Satb2 in the mouse cerebral cortex and hippocampus resulted in hyperactivity, increased impulsivity, abnormal social novelty, and impaired spatial learning and memory. Thus, our Satb2 CKO mice may serve as a mouse model for studying the underlying mechanism of the SAS associated with Satb2 mutation in patients.

## Materials and Methods

### Experimental Animals

Animal care practices and all experiments were reviewed and approved by the Animal Committee of Tongji University School of Medicine, Shanghai, China. We used Satb2-targeted embryonic stem cells (EPD0098_3_H05), purchased from the International Mouse Phenotyping Consortium, to generate the Satb2 knockout-first mice, which were initially crossed with FLPeR mice to obtain floxed Satb2 mice. To delete Satb2 in the cerebral cortex and hippocampus, Emx1-Cre mice (Guo et al., 2000) were crossed with Satb2^flox/flox^ mice to obtain Emx1-Cre; Satb2^flox/flox^ mice (Satb2 CKO) mice. In the offspring, these genotypes (i.e., Satb2^flox/+^ or Satb2^flox/flox^) were used as controls.

### Immunohistochemistry, AuCl_3_ Staining and *in situ* Hybridization

Mice were perfused with 4% paraformaldehyde (PFA) at different postnatal ages. All brains were fixed in 4% PFA overnight, cryoprotected in 30% sucrose in phosphate-buffered saline overnight and cut into 20 μm-thick sections. For immunohistochemistry, brain sections were incubated with rabbit anti-Satb2 (1:300, Abcam) or goat anti-5-HTT antibody (1:1000, Immunostar) at 4°C overnight, and then incubated with biotinylated horse anti-rabbit IgG or horse anti-goat IgG (1:500, Jackson ImmunoResearch) at room temperature for 3 h followed by incubation with streptavidin-Cy3 (1:1000, Jackson ImmunoResearch) and counterstaining with Hoechst 33258 (1:2000, Sigma) at room temperature for 1 h.

The AuCl_3_ staining was performed as a previous study (Wahlsten et al., 2003). The brain sections were stained with 0.2% gold chloride (AuCl_3_) in phosphate buffer. The process was taken place in darkness. Once axonal staining became evident, the reaction was stopped by transferring sections to 2.5% sodium thiosulfate anhydrous for 5 min.

The antisense digoxigenin-labeled RNA probes of RORβ, Cux2, Ctip2, and Tle4 were synthesized according to the Allen Brain Atlas website, and *in situ* hybridization was performed as described in our previous study (Song et al., 2011).

### Behavioral Tests

Adult (3–6 months old) male mice were used in the following behavioral tests. All behavioral experiments were performed during the light phase of the light/dark cycle. Behavioral tests were conducted in a sound-proof room with a neutral environment. All mice were given a 30-min habituation time after transport to the behavioral test room. There were 2 or 3 days for resting between different tests. The experimenter was blind to the group identity of the tested mice. Some behavioral tests were recorded with a camera and a trained researcher analyzed these videos.

#### Open Field Test

The open field apparatus comprised a square arena, with a white floor divided into 9 squares (10 cm × 10 cm) and enclosed by continuous 21 cm-high walls made of transparent plexiglass. The experiment lasted for 30 min. Average velocity, total distance traveled, ambulatory time, and average velocity were recorded by Activity Monitor software (Med Associates, St. Albans, VT, United States).

#### Cliff Avoidance Reaction

The cliff avoidance reaction (CAR) is based on the natural tendency of animals to avoid a potential fall from a height (Yamashita et al., 2013). The apparatus used in the CAR test included a round wooden platform (diameter, 20 cm) supported by a heavy rod (height, 50 cm). Two identical apparatus were used for the test. The test was initiated by placing mice on a platform such that the forelimbs approached its edge. If the mouse fell from the platform, it was immediately placed back on the platform and was considered to have impaired CAR. The experiment lasted for 30 min. The latency from the initial placement on the platform until falling was recorded. The incidence of impaired CAR was calculated as a percentage index for each group, as follows:

% (CAR) = [the number of mice that did not fall from the platform/total numbers of tested mice] × 100.

#### Dark-Light Exploration Test

This test was performed to assess the anxiety-like behaviors of rodents, as described in our previous study (Zhang et al., 2016). The apparatus was a rectangular plexiglass box (45 cm length × 20 cm width × 20 cm height) divided into a smaller (1/3) black area with a lid and a larger (2/3) white area with an open-top. A black wall separated the two compartments and had an opening door (5 cm × 5 cm) at floor level. The light intensity was about 500 lx in the white part. Each mouse was placed in the center of the dark compartment and behavior was recorded over a 5-min period. The time spent in the white box and the number of transitions between dark and white compartments were recorded.

#### Elevated Plus-Maze Test

This test assesses anxiety-like behaviors in rodents, as described in our previous study (Zhang et al., 2016). The elevated plus-maze consisted of two open arms (30 cm × 5 cm), two enclosed arms (30 cm × 5 cm), and a central platform (5 cm × 5 cm). The maze was elevated 40 cm above the ground. Each mouse was placed in the central platform facing one of the enclosed arms and was observed for 5 min. The time spent in the open arms and the number of entries into the open arms were recorded. Open arm entry was defined as a mouse having entered an open arm with all four legs.

#### Pre-pulse Inhibition Test

The mouse was subjected to a pre-pulse inhibition (PPI) test in a startle chamber (SR-LAB; San Diego Instruments, San Diego, CA, United States) using the standard methods described previously (Geyer and Dulawa, 2003). The test sessions were started after an initial 5-min acclimation period in the chamber. Each PPI test session comprised 64 trials. Mice were subjected to one of the following five trials: (1) pulse alone, as a 40-ms burst (120 dB); a 40-ms pulse burst preceded by 100 ms with a 20-ms pre-pulse that was (2) 5 dB, (3) 13 dB, or (4) 22 dB over background (60 dB), namely, pre-pulse + pulse trials; and (5) background only (no-stimulus). Each test session began and ended with six presentations of the pulse-alone trial; between these, pre-pulse + pulse and no-stimulus trials were presented 10 times each, and the pulse-alone trials 12 times each, and in a pseudorandom order. The inter-trial interval was 7–23 s (15 s on average). The initial and final six pulse-alone trials were not included in the analysis. The amount of PPI was expressed as the percentage decrease in the amplitude of the startle reactivity caused by presentation of the pre-pulse, which was calculated as follows: % PPI = 100 - {[(startle response for pre-pulse + pulse)/(startle response for pulse alone)] × 100}.

#### Three-Chamber Test

The three-chamber social test is an accepted and sensitive measure of social behavior in mice. The apparatus is a rectangular plexiglass box (90 cm length × 50 cm width × 30 cm height) divided into three equal chambers. Mice are allowed to access each compartment by crossing the door, which is a square opening (5 cm × 5 cm) located at floor level of the partition. Two inverted wire-mesh cylinders were placed at the corners of the two side chambers and a weighted bottle was placed on the top of the cylinders to prevent the animal from climbing on the top of them. The test was performed as described previously (Zhang et al., 2016) with minor modifications. The day before the test, all test mice were habituated to the apparatus for 20 min with the two empty cylinders inside, and all stranger mice were separately habituated inside the wire cylinders for 20 min at a time. On the test day, after a 10 min habituation period, all mice were tested in two conditions. In the first condition, an unfamiliar sex- and age-matched C57BL/6J mouse (stranger 1, S1) was placed in one cylinder and an inanimate ball was placed in the other cylinder. The test mouse was placed in the middle chamber and was allowed to explore the three chambers for 10 min. In this phase (sociability phase), the test mouse had the choice to sniff the unfamiliar mouse (S1) or a novel object (ball). Ball and S1 preference, respectively, were calculated as follows: Ball preference % = [time spend to explore ball/(time spent exploring the ball + time spent to interact with S1)] ^∗^ 100; S1 preference % = [time spent interacting with S1/(time spent to explore ball + time spent to interact with S1)] ^∗^ 100. In the second condition, a novel mouse (stranger 2, S2) replaced the inanimate ball. The test mouse was then placed back into the middle chamber and was again allowed to explore the three chambers for 10 min. Thus, the test mouse had the choice to interact with S1 or S2 in this social novelty phase. S1 and S2 preference, respectively, were calculated as follows: S1 preference % = [time spent to interact with S1/(time spent to interact with S1 + time spent to interact with S2)] ^∗^ 100; S2 preference % = [time spent to interact with S2/(time spent to interact with S1 + time spent to interact with S2)] ^∗^ 100. Interaction time recordings began when test mice sniffed within 2 cm of the cages. The location of S1, S2 and the ball were changed between tests.

#### Direct Interaction Test

The direct interaction test was designed as described previously (Zhang et al., 2016), with some modifications. The experimental apparatus is a non-transparent, open-topped box (45 cm × 45 cm × 45 cm). One day before the test, all test mice and stranger mice were habituated to the arena for 20 min. On the test day, test mice were put in the box for a 5-min habituation period, and then a novel stimulus C57BL/6 J mouse (age- and sex-matched) was brought into the same box for 10 min. The following behaviors were recorded as social interaction: anogenital and nose-to-nose sniffing, following (within 2 cm), and allogrooming. Any aggressive behaviors between animals led to termination of the experiment and exclusion of the data from the analysis.

#### Morris Water Maze Test

The Morris water maze (MWM) test was used to evaluate spatial learning and memory in rodents. The test used a 1.2-m diameter circular blue pool, which was divided into four hypothetical, equal quadrants. A hidden circular platform (11 cm diameter) located in the middle of the target quadrant was submerged approximately 1.5 cm beneath the surface of the water. In this test, mice need to navigate to the hidden platform using spatial cues on the surrounding area across multiple trials. First, mice were trained to find the hidden platform during the learning phase. For this, four trials were conducted per day for 7 consecutive days. On the 8th day, the platform was removed and each mouse was allowed 60 s to search the pool for the platform. Noldus software (EthoVision XT 8.0, Noldus Technology) was used to monitor and track the movement of mice. Latency to find the platform, mean distance to platform, frequency of platform crosses, duration spent in the target quadrant, and total distance traveled were measured automatically by the software.

### Statistical Analysis

Statistical analyses were performed using IBM SPSS Statistics 19 software. Differences in weight between three groups (controls, Satb2 heterozygotes and Satb2 CKO mice) were analyzed by one-way ANOVA test. Differences between Satb2 CKO and control mice in the open field, dark-light exploration test, elevated plus-maze test, three-chamber test, direct interaction test and test phase of the MWM test were analyzed by Student’s *t*-tests. Between genotype effects during the PPI test and the acquisition phase of the MWM task were analyzed by a repeated measures ANOVA, followed by a least significant difference test with genotype and pre-pulse intensities value, where genotype and days were the factors, respectively. Results were considered significant when *P*-value < 0.05.

## Results

### Generation of Satb2 CKO Mice by Deletion of Satb2 in the Cerebral Cortex and Hippocampus

**FIGURE 1:**
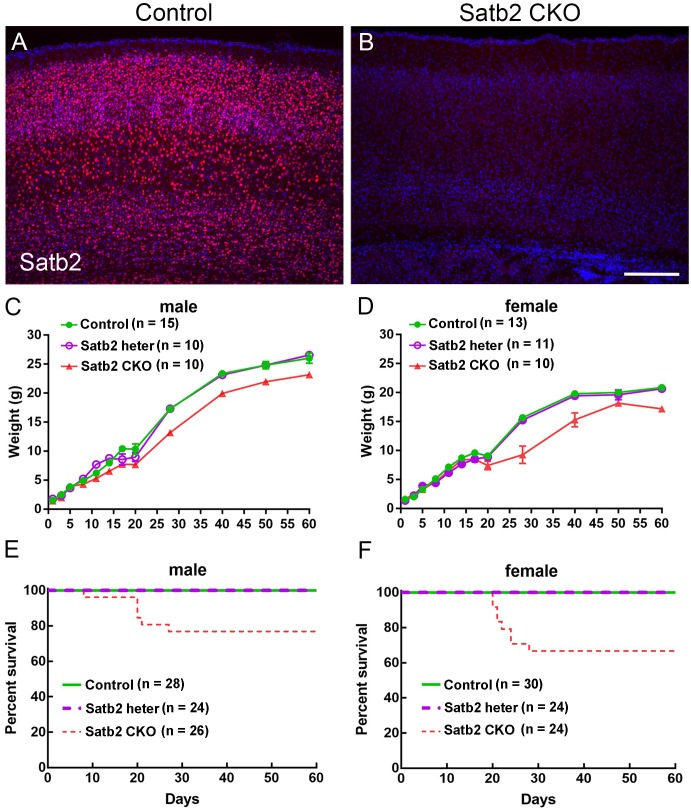
Reduced body weight and survival rate in adult Satb2 CKO mice. **(A,B)** Satb2 immunostaining verified that Satb2 was deleted in the cerebral cortex in Satb2 CKO mice. **(C,D)** The growth curve of male **(C)** and female **(D)** control, Satb2 heterozygotes, and Satb2 CKO mice. Both male and female Satb2 heterozygotes (Emx1-Cre; Satb2^flox/+^) and CKO mice had a normal body weight until postnatal day 15 (P15). The one-way ANOVA test measured the significant differences between these groups appeared at P15 in male mice {*F*[2,32] = 14.55, *P* < 0.0001; Tukey’s multiple comparisons test showed *P* = 0.7673 (controls vs. Satb2 heterozygotes); *P* < 0.0001 (controls vs. Satb2 CKO mice); *P* = 0.0008 (Satb2 heterozygotes vs. Satb2 CKO mice)}, and P20 in female mice {*F*[2,31] = 4.697, *P* = 0.0165; Tukey’s multiple comparisons test showed *P* = 0.9858 (controls vs. Satb2 heterozygotes); *P* = 0.0225 (controls vs. Satb2 CKO mice); *P* = 0.0406 (Satb2 heterozygotes vs. Satb2 CKO mice)}. **(E,F)** The survival rate of male **(E)** and female **(F)** wild-type, Satb2 heterozygotes, and Satb2 CKO mice. The survival rate of female Satb2 CKO mice was lower than male CKO mice. In **(C,D)**, data are presented as the mean ± SEM. Scale bar = 200 μm **(B)**.

Our previous study has shown that Satb2 is abundantly expressed in the cerebral cortex and hippocampus (Huang et al., 2013a). To delete Satb2 in these two regions, we crossed Emx1-Cre mice (Guo et al., 2000) with Satb2^flox/flox^ mice to obtain Emx1-Cre; Satb2^flox/flox^ (Satb2 CKO) mice. Littermates with other genotypes (i.e., Satb2^flox/+^ and Satb2^flox/flox^) showed no alterations examined below and were used as control mice. Deletion of Satb2 in the cerebral cortex of CKO mice was confirmed by immunostaining (Figure 1A,B). As Cre recombinase is exclusively active in the brain of Emx1-Cre mice (Guo et al., 2000), the cleft palate developed normally in Satb2 CKO mice (data not shown). Satb2 CKO mice showed normal body weight at birth (Figure 1C,D), but there was about a 20% reduction in body weight at adulthood compared with age-matched control mice (Figure 1C,D). The reduced body weight was first observed at around P15 in male and P20 in female Satb2 CKO mice (Figure 1C,D). Unlike conventional Satb2 mutant mice, which died at birth (Alcamo et al., 2008; Britanova et al., 2008), all the Satb2 CKO mice survived after birth and about 2/3 of CKO mice survived into adulthood (Figure 1E,F). Nevertheless, it is clear that Satb2 deletion in the cerebral cortex and hippocampus leads to growth retardation, which is present in patients with SAS (Zarate and Fish, 2017).

### Hyperactivity and Increased Impulsivity in Satb2 CKO Mice

**FIGURE 2:**
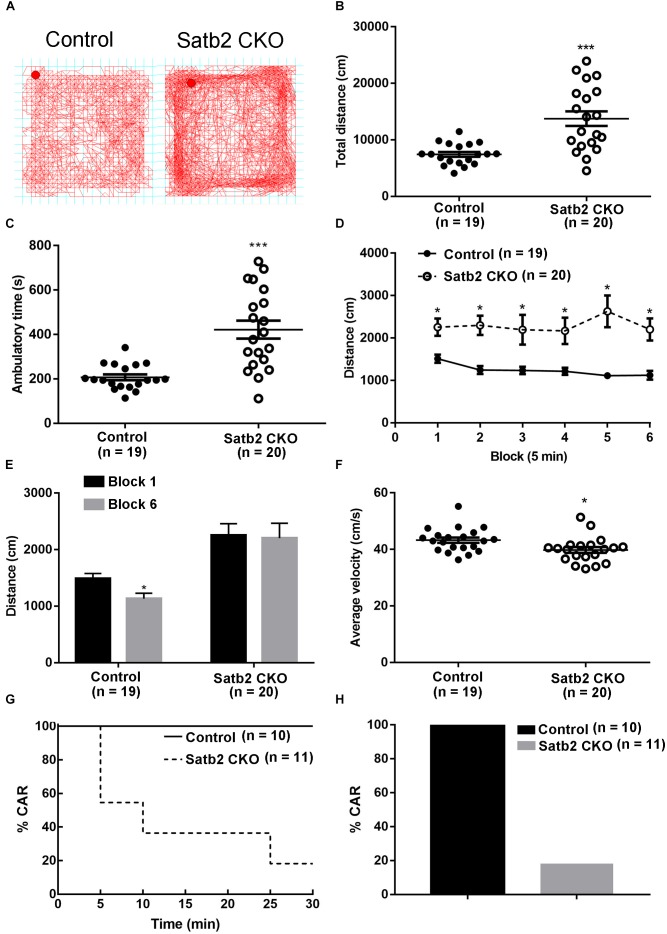
Hyperactivity and increased impulsivity in Satb2 CKO mice. **(A)** The traveling trace in the open field of the control and Satb2 CKO mice. **(B)** The total distance traveled by Satb2 CKO mice in the open field was much higher than that of control mice (*t*[37] = 4.553, *P* < 0.001). **(C)** The ambulatory time of Satb2 CKO mice was longer than that of the control mice in the open field (*t*[37] = 4.951, *P* < 0.001). **(D,E)** The distance traveled was analyzed every 5 min; it gradually reduced in control mice over time, whereas this was not observed in Satb2 CKO mice as shown by persistent moving in the field **(D)**. The traveled distance in block 6 was significantly lower than that in block 1 in control mice and comparable with that in block 1 in Satb2 CKO mice (**E**, *t*_control_[36] = 2.687, *P* = 0.0108; *t*_CKO_[38] = 0.1505, *P* = 0.8812). **(F)** The average velocity of Satb2 CKO mice was lower than that of control mice (*t*[37] = 2.196, *P* = 0.0345). **(G,H)** The CAR test was performed to test impulsivity. During the 30-min test, more Satb2 CKO mice fell from the platform than did control mice, none of which fell **(G)**. In total, about 80% of Satb2 CKO mice fell from the platform **(F)**. In **(B–F)**, data are presented as the mean ± SEM; each dot represents a mouse in **(B,C,F)**. ^∗^*P* < 0.05, ^∗∗∗^*P* < 0.001 via Student’s *t*-tests in **(B,C,E,F)**. Data were analyzed using a repeated measures ANOVA in **(D)**.

We noticed that Satb2 CKO mice showed hyperactivity in their home cages. To further examine locomotor activity, the open-field test was performed. We found that the total distance traveled was significantly greater in Satb2 CKO mice relative to controls (Figure 2A,B) and the ambulatory time of Satb2 CKO mice was also greater compared with that of control mice (Figure 2C). These results indicated that hyperactivity was present in the Satb2 CKO mice.

To examine the phenotype of hyperactivity in more detail, we analyzed activity in the open field test every 5 min. The distance traveled gradually reduced in control mice, whereas it showed no obvious change in Satb2 CKO mice over time (Figure 2D,E). Although the total distance traveled was greater in Satb2 CKO mice compared to controls, the average velocity was less than that of control mice (Figure 2F). Hyperactivity is also observed in patients with attention-deficit/hyperactivity disorder (ADHD), which has a characteristic locomotor-related symptom called impulsive behavior (Willcutt, 2012). The cliff avoidance reaction (CAR) is widely used to assess impulsive behavior in rodents (Yamashita et al., 2013). During the 30-min test, about 80% of Satb2 CKO mice fell from the platform, whereas no control mice did so (Figure 2G,H). Taken together, Satb2 CKO mice showed hyperactivity and impulsivity behaviors.

### Reduced Anxiety-Like Behaviors in Satb2 CKO Mice

**FIGURE 3:**
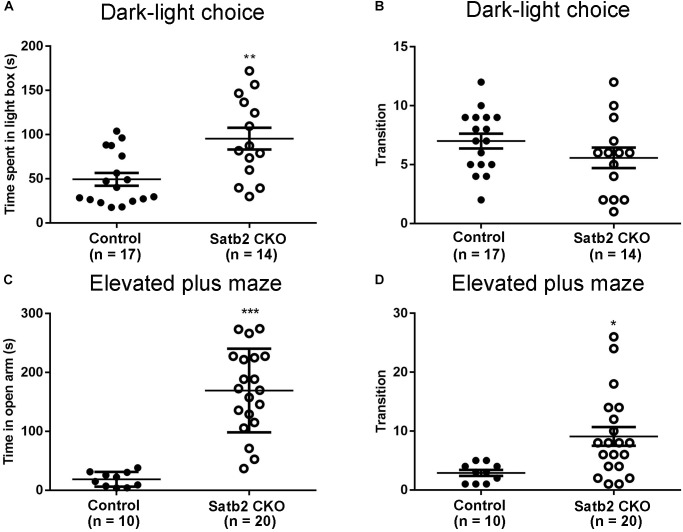
Reduced anxiety-like behaviors in Satb2 CKO mice. **(A,B)** The dark-light choice test showed that Satb2 CKO mice spent more time in the light box than did control mice (**A**, *t*[29] = 3.362, *P* = 0.0022). The transition number of Satb2 CKO mice was comparable with that of control mice (**B**, *t*[29] = 1.361, *P* = 0.1841), probably due to more time spent in the light box. **(C,D)** The elevated plus maze test showed that the time Satb2 CKO mice spent in the open arms was significantly longer than that of control mice (**C**, *t*[28] = 6.597, *P* < 0.0001). Satb2 CKO mice exhibited a higher transition number than control mice (**D**, *t*[28] = 2.699, *P* = 0.0116). In **(A–D)**, data are presented as the mean ± SEM and each dot represents a mouse. ^∗^*P* < 0.05, ^∗∗^*P* < 0.01, ^∗∗∗^*P* < 0.001 via Student’s *t*-tests.

The anxiety-like behaviors were examined in Satb2 CKO mice. First, the dark-light choice test showed that Satb2 CKO mice spent more time in the light box than did control mice (Figure 3A). However, the transition number of Satb2 CKO mice was comparable with control mice (Figure 3B), which may be a consequence of spending more time in the light box. Second, the elevated plus maze test showed that Satb2 CKO mice spent more time in the open arms than did control mice (Figure 3C). Satb2 CKO mice also exhibited more arm transitions than the control mice (Figure 3D). These data indicate reduced anxiety-like behaviors in Satb2 CKO mice.

### Abnormal Sensorimotor Gating and Social Interaction in Satb2 CKO Mice

**FIGURE 4:**
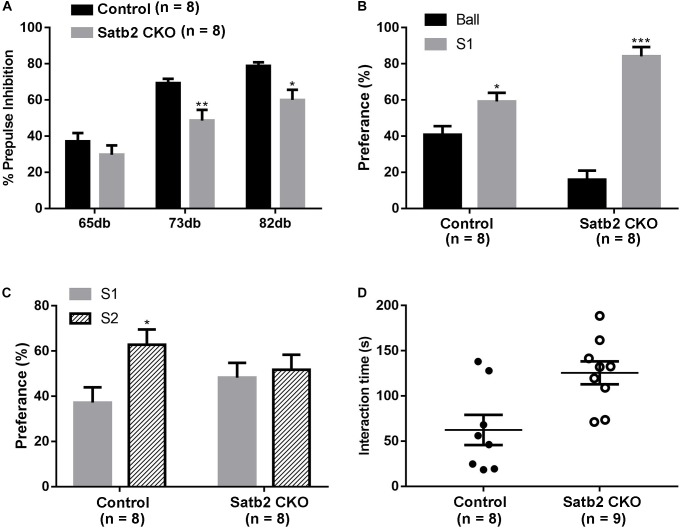
Impaired sensorimotor gating and social novelty in Satb2 CKO mice. **(A)** The pre-pulse inhibition (PPI) test shows that Satb2 CKO mice had a significant PPI deficit compared with control mice at 73 and 82 dB pre-pulse intensities. The repeated measures ANOVA (2 genotypes × 3 pre-pulse intensities with repeated measures on pre-pulse intensities) showed that both the genotypes and pre-pulse intensities affected response reactivity {genotypes effect: *F*[1,42] = 17.76, *P* = 0.0001; pre-pulse intensities effect: *F*[2,42] = 33.52, *P* < 0.0001; interaction: *F*[2,42] = 1.246, *P* = 0.298; *P* = 0.0074 (73dB); *P* = 0.0165 (82 dB); *P* = 0.5856 (65 dB)}. **(B,C)** The three-chamber social interaction test was performed. Satb2 CKO mice showed a preference for the animate stranger mouse (S1) over the inanimate ball, with no difference compared with control mice (**B**, *t*_control_[14] = 2.755, *P* = 0.0155; *t*_CKO_[14] = 9.409, *P* < 0.0001). Satb2 CKO mice showed a similar preference to the S2 mouse and S1 mouse, while the control mice showed a preference for the S2 mouse (**C**, *t*_control_[14] = 2.673, *P* = 0.0182; *t*_CKO_[14] = 0.3878, *P* = 0.7040). **(D)** In the direct social interaction test, the interaction time of Satb2 CKO mice was longer compared with control mice (*t*[15] = 3.057, *P* = 0.0080). In **(A–D)**, data are presented as the mean ± SEM and each dot represents a mouse in **(D)**. ^∗^*P* < 0.05 via Student’s *t*-tests in **(B–D)**, and repeated measures ANOVA in **(A)**. ^∗∗^*P* < 0.01, ^∗∗∗^*P* < 0.001.

Pre-pulse inhibition of the acoustic startle response test, which is a measure of sensorimotor gating, was performed. The Satb2 CKO mice showed significant PPI deficits compared with control mice at 73 and 82 dB pre-pulse intensities, and showed an increased response tendency at 65 dB intensities (Figure 4A). The abnormal PPI reaction in Satb2 CKO mice indicates that the sensorimotor gating is disturbed in Satb2 CKO mice.

The social-related behaviors was then tested in the control and Satb2 CKO mice. The three-chamber social interaction test was first performed. Both control mice and Satb2 CKO mice showed a preference for S1 over the inanimate ball (Figure 4B), which indicates normal social ability in Satb2 CKO mice. In the social novelty phase, Satb2 CKO mice showed a similar preference for S2 and S1 mice, while the control mice showed a preference for the S2 mouse (Figure 4C), suggesting that social novelty is impaired in Satb2 CKO mice. In the direct social interaction test, however, Satb2 CKO mice spent more time interacting with the stranger mouse than did the control mice (Figure 4D). Taken together, these results indicate that sensorimotor gating is impaired and social interaction behaviors are altered in Satb2 CKO mice.

### Defective Spatial Learning and Memory in Satb2 CKO Mice

**FIGURE 5:**
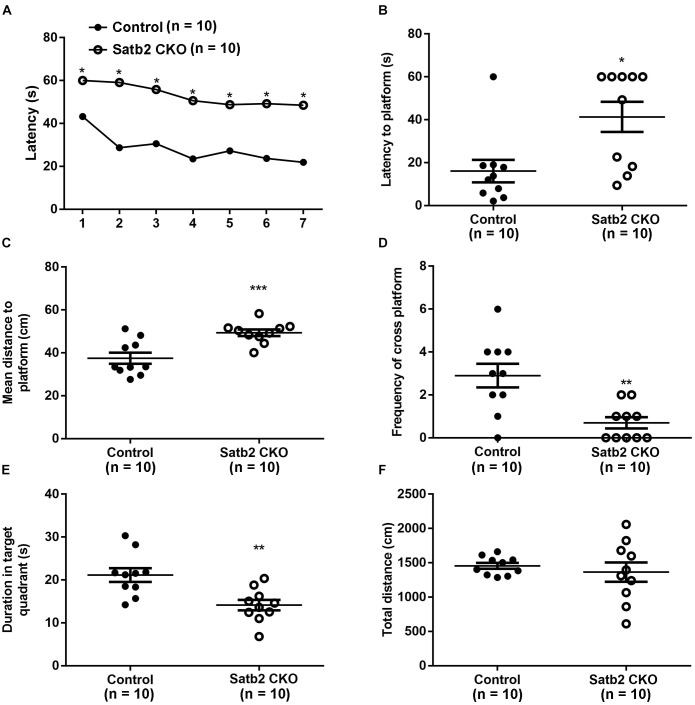
Impaired spatial learning and memory in Satb2 CKO mice. The Morris water maze test was performed. **(A)** During the learning phase, the latency to the platform was significantly longer in Satb2 CKO mice than in the control mice. The analysis of the latency using a repeated measures ANOVA (2 genotypes × 7 days with repeated measures on days) showed that both the genotypes and training days affected learning ability (genotype effect: *F*[1,18] = 38.53, *P* < 0.0001; days effect: *F*[6,108] = 11.28, *P* < 0.0001; and interaction: *F*[6,108] = 1.59, *P* = 0.1571). **(B)** During the memory trial, the latency to the platform was longer in Satb2 CKO mice compared with the control mice (*t*[18] = 2.872, *P* = 0.0101). **(C)** During the memory trial, the mean distance to the platform was higher in the Satb2 CKO mice compared with the control mice (*t*[18] = 3.944, *P* = 0.0010). **(D)** The duration in the target quadrant was shorter in Satb2 CKO mice compared to control mice (*t*[18] = 3.493, *P* = 0.0026). **(E)** The frequency of platform crossings was lower in Satb2 CKO mice compared to control mice (*t*[18] = 3.633, *P* = 0.0019). **(F)** Satb2 CKO mice showed a similar swimming velocity to control mice (*t*[18] = 0.6638, *P* = 0.5153). In **(A)**, data are presented as the mean and ^∗^*P* < 0.05 by a repeated measures ANOVA. In **(B–F)**, data are presented as the mean ± SEM, and each dot represents a mouse. ^∗^*P* < 0.05, ^∗∗^*P* < 0.01, ^∗∗∗^*P* < 0.001 via Student’s *t*-tests.

Deletion of Satb2 in the hippocampus with CamKII-Cre at the postnatal stage leads to impairment of long-term fear memory and object recognition memory (Jaitner et al., 2016). We examined whether spatial learning and memory was altered in the CKO mice, in which Satb2 was deleted in the cortex and hippocampus at the embryonic stage. During the learning phase of the MWM, Satb2 CKO mice exhibited a longer latency in finding the platform than control mice (Figure 5A). The net decrease of the latency between days 1 and 7 in the control and Satb2 CKO mice were 21.33 ± 3.589 s and 9.944 ± 4.138 s, respectively. These results indicated that the Satb2 CKO mice may have impaired learning capacity during the consecutive 7-days training phase of the MWM task than their control counterparts. During the memory test, the latency to the location of platform and the mean distance to the platform was longer in Satb2 CKO mice compared with those in the control mice (Figure 5B,C). Consistently, the number of platform crossings and the duration spent in the target quadrant were lower in the Satb2 CKO mice compared to control mice (Figure 5D,E). These observed changes were not due to the different swimming abilities, as shown by the similar swimming distances between Satb2 CKO and control mice (Figure 5F). Thus, Satb2 CKO mice showed impaired spatial learning and memory.

### Loss of “Barrels” in Layer IV in the Cerebral Cortex of Satb2 CKO Mice

**FIGURE 6:**
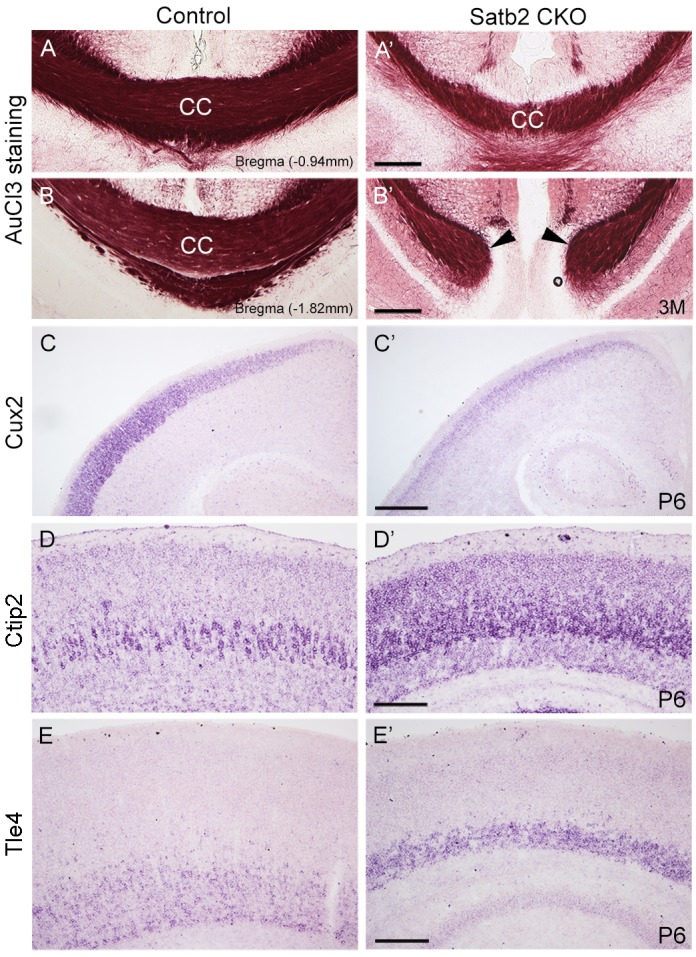
Altered corpus callosum and gene expression in the cortex of Satb2 CKO mice. **(A–B^′^)** A few of AuCl_3_ labeled axons cross the midline at anterior section **(A^′^)** but not posterior section **(B^′^)** in Satb2 CKO mice compared with control mice **(A,B)**. **(C,C^′^)** Cux2 mRNA was dramatically decreased in layers II–IV at P6 in Satb2 CKO mice **(C^′^)** compared with control mice **(C)**. **(D,D^′^)** Ctip2 mRNA was increased in Satb2 CKO cortex **(D^′^)** compared with the control mice **(D)**. **(E,E^′^)** Tle4 mRNA was increased in VI of Satb2 CKO mice **(E^′^)** when compared with control mice **(E)**. Scale bar = 500 μm **(C^′^)**, 200 μm **(A^′^,B^′^,D^′^,E^′^)**.

**FIGURE 7:**
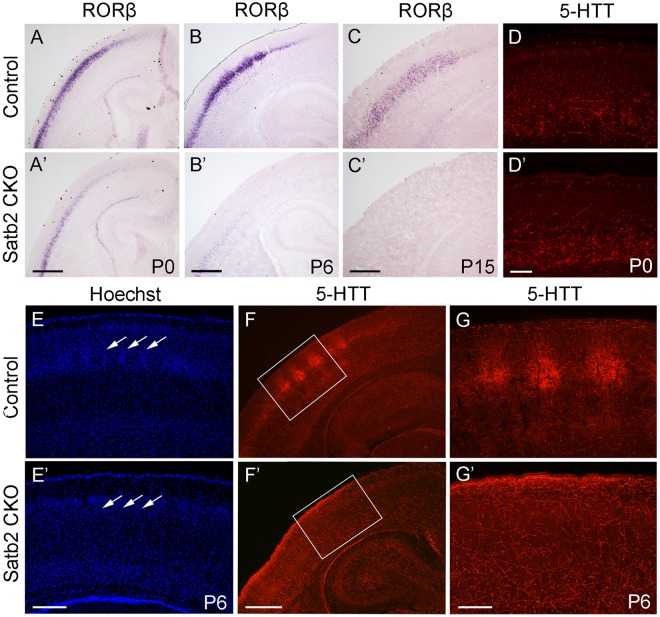
Loss of “barrels” in layer IV in Satb2 CKO mice. **(A,A^′^)** RORβ mRNA was dramatically reduced in the cortex at P0 in Satb2 CKO mice **(A^′^)** compared with that in control mice **(A)**. **(B–C^′^)** The expression of RORβ mRNA was hardly detected in Satb2 CKO mice at P6 **(B^′^)** and totally absent at P15 **(C^′^)**. **(D,D^′^)** 5-HTT-positive fibers reached the deep layer of cortex in Satb2 CKO mice **(D^′^)** at P0 as control mice did so **(D)**. **(E,E^′^)** Septal regions among the “barrels” can be clearly seen in a control mouse, as shown by the presence of densely packed Hoechst-stained cells (**E**, arrows), but were not observed in Satb2 CKO mice, as shown by homogenous distribution of stained cells (**E^′^**, arrows). **(F–G^′^)** Thalamocortical axons labeled by 5-HTT in layer IV were clustered within “barrels” in control mice **(F,G)**, while they were sparsely and homogenously distributed in layer IV of Satb2 CKO mice **(F^′^,G^′^)**. Scale bar = 500 μm **(A^′^–C^′^,F^′^)** and 100 μm **(D^′^–E^′^,G^′^)**.

In the morphogenesis of the cerebral cortex, Satb2 is known to regulate the development of both callosal projection neurons in layers II–III (Alcamo et al., 2008; Britanova et al., 2008) and subcerebral projection neurons in layer V (Leone et al., 2015; McKenna et al., 2015). We first examined if the corpus callosum is affected in Satb2 CKO mice. As shown in Figure 6A,A^′^, although AuCl_3_-labeled callosal axons were present in the midline region at the level around Bregma -0.94 mm in Satb2 CKO mice, their thickness was reduced relative to the control. At caudal level around Bregma -1.82 mm, AuCl_3_-labeled corpus callosum was present in the control mice (Figure 6B) but absent in Satb2 CKO mice (arrowheads, Figure 6B^′^). Next, we moved to examine the expression of layer-specific genes in Satb2 CKO mice. Cux2 was expressed in layers II–IV in the control mice (Figure 6C), but its expression was much reduced in Satb2 CKO mice (Figure 6C^′^). Consistent with previous studies (Alcamo et al., 2008), Ctip2 was strongly expressed in layer V, but intense Ctip2 expression expanded into layers II–IV in Satb2 CKO mice at P6 (Figure 6D,D^′^). Tle4 is one of the deep layer markers, and mainly expressed in layer VI (Figure 6E) (Bin et al., 2005). However, Tle4 expression was increased in Satb2 CKO mice at P6 (Figure 6E^′^). Next, we examined whether the development of layer IV cortical neurons is affected in Satb2 CKO mice. RORβ is a specific marker for layer IV cortical neurons (Takeuchi et al., 2007) and we found that RORβ mRNA was dramatically reduced at P0 (Figure 7A,A^′^), more severely reduced at P6 (Figure 7B^′^), and totally lost in the Satb2 CKO mice at P15 (Figure 7C^′^). In addition, layer IV is the main target area for thalamocortical projections, and layer IV neurons together with thalamocortical projection axons, particularly those relaying sensory information from the whiskers, form “barrels” in the somatosensory cortex in rodents (Woolsey, 1990; Ding et al., 2003). The dense distribution of Hoechst-stained cells in the septal regions revealed the “barrels” in the control mice (arrows, Figure 7E). However, barrels were absent in Satb2 CKO mice, as shown by the homogenous distribution of Hoechst-stained cells in the somatosensory cortex (arrows, Figure 7E^′^). Meanwhile, the serotonin transporter (5-HTT) expressed by thalamocortical projection axons were densely located within individual barrels in layer IV of the wild-type somatosensory cortex (Figure 7F,G), but 5-HTT-positive axons were sparsely and homogenously distributed in layer IV of Satb2 CKO mice (Figure 7F^′^,G^′^), further supporting the loss of the somatosensory map in the cortex of Satb2 CKO mice. Furthermore, we examined whether the process of thalamocortical axons entering cortex is affected in Satb2 CKO mice. 5-HTT-positive fibers reached the deep layer of cortex at P0 in Satb2 CKO mice as the control mice did (Figure 7D,D^′^). It should be noted that 5-HTT-positive fibers did not aggregate in layer IV in both control mice and Satb2 CKO mice at this stage (Figure 7D,D^′^). Previous studies have revealed impaired development of cortical neurons in layers II–III and V in the absence of Satb2 (Alcamo et al., 2008; Britanova et al., 2008). Furthermore, we demonstrated that the development of layer IV cortical neurons and the inputs from the thalamus are also impaired.

## Discussion

Satb2-associated syndrome, caused by the alteration in the Satb2 gene, is characterized by growth delay, intellectual disability, abnormal behaviors, and craniofacial and skeletal anomalies (Zarate and Fish, 2017). To investigate the fundamental basis of SAS-associated intellectual disability and abnormal behaviors, and explore the behavioral consequences of Satb2-implicated behaviors, Satb2 was specifically deleted in the cerebral cortex and hippocampus in mice by crossing Emx1-Cre with Satb2^flox/flox^ mice. Unlike Satb2 KO and previous Satb2 CKO mice, which died at birth and juvenile period, respectively (Alcamo et al., 2008; Leone et al., 2015), about 2/3 of CKO mice in this study survived into adulthood. We found that the Satb2 CKO mice exhibited hyperactivity, increased impulsivity, reduced anxiety-like behaviors, defective sensorimotor gating, and abnormal social interaction behaviors, which reflect most of the behavioral abnormalities observed in individuals with SAS.

Previous case reports have consistently reported the presence of developmental delay/intellectual disability in individuals with SAS (Zarate et al., 2017, 2018). In previous research, CamKIIα-Cre;Satb2 CKO mice with postnatal deletion of Satb2 showed impairments in long-term memory in a contextual fear conditioning test and object location memory test (Jaitner et al., 2016), and in spatial learning and memory in the MWM test (Li et al., 2017). Our Satb2 CKO mice also displayed impaired spatial learning and memory (Figure 5). Furthermore, previous work has found that a deletion of Satb2 via AAV-Cre viral delivery to the hippocampus of adult Satb2^flox/flox^ mice also leads to impaired spatial learning and memory (Li et al., 2017). Thus, loss of Satb2 in the hippocampus alone is sufficient to disturb spatial learning and memory in mice.

It has been reported that over 80% of adult individuals with SAS have a jovial personality (Zarate et al., 2018). Although there are no standard behavioral paradigms to analyze this in mouse models of SAS, anxiety-like behaviors can, to some extent, reflect emotional states; we found reduced anxiety-like behaviors in Satb2 CKO mice compared to control mice (Figure 3). In addition, more than 20% of individuals with SAS show hyperactivity and distractibility (Zarate et al., 2017, 2018), which are also symptoms of ADHD. Satb2 CKO mice were overactive in their home cages compared with the control mice, and exhibited hyperactivity in the open field (Figure 2). In the CAR test, Satb2 CKO mice were prone to falling from the platform, which may be caused by distractibility (Figure 2). Furthermore, about 20% of individuals with SAS exhibit autistic behaviors (Zarate et al., 2017, 2018). Using the three-chamber test and direct social interaction test, we found that Satb2 CKO mice showed normal social abilities; however, social novelty was disturbed (Figure 4). Finally, about 10% of individuals with SAS have social affective behaviors and sensory issues (Zarate et al., 2018), and Satb2 has been recognized as a risk gene for schizophrenia (Jaitner et al., 2016). Consistent with these findings, Satb2 CKO mice showed significant and distinct PPI deficits compared to control mice. Thus, most symptoms in patients with SAS were observed in our mouse model with the deletion of Satb2 in the cerebral cortex and hippocampus at embryonic stages, and it may therefore serve as an animal model for studying the neurobiological mechanisms underlying SAS.

Satb2 is a determinant gene for the cortical neuronal fate in layers II–III and layer V–VI (Alcamo et al., 2008; Britanova et al., 2008; Leone et al., 2015; McKenna et al., 2015). Consistent with results from a previous study (Alcamo et al., 2008), we confirmed RORβ mRNA was dramatically reduced at P0, more severely reduced at P6, and totally lost at P15 in Satb2 CKO mice (Figure 7). In addition, loss of “barrels” in layer IV of the somatosensory cortex was found in Satb2 CKO mice. The formation of this unique structure is driven by whisker-related sensory inputs carried by thalamocortical projections during the early postnatal period (Killackey et al., 1990; Huang et al., 2013b). The loss of this somatosensory map strongly suggests that sensory information, including whisker-related information, cannot be processed properly within the cerebral cortex after deletion of Satb2. In addition, the corpus callosum is responsible for communication between the two hemispheres and therefore important for higher brain functions (Paul et al., 2007). In previous studies, Satb2 KO and Satb2 CKO mice have shown impairment of callosal development, with reduced axon projections across the midline (Alcamo et al., 2008; Leone et al., 2015). A similar phenotype has been observed in our Satb2 CKO mice. The corpus callosum was reduced at the anterior level and absent at the caudal level in Satb2 CKO mice (Figure 6). Data from gene expression and axonal connections demonstrate that the development of cortical neurons in multiple layers is impaired, and these defects may lead to behavioral abnormalities in Satb2 CKO mice. Besides, a large number of genes are differentially expressed in Satb2 CKO mice, and some of them are risk genes associated with schizophrenia and other neurodevelopmental disorders (Whitton et al., 2018). Moreover, the cortical layers II-III contain the major population of callosal neurons (Wang et al., 2007), and their neuronal identities are severely affected in Satb2 CKO mice. Importantly, the excess of pyramidal neurons in cortical layers II-III caused autism-like behaviors in a mouse model (Fang et al., 2014). Thus, Satb2 may play some roles in the pathogenesis of these neurodevelopment-associated mental diseases.

## Author Contributions

QZ and YH conception and design, data acquisition, analysis and interpretation, and drafting and revising the article. LZ, N-NS, and Y-QD conception and design, data analysis and interpretation, supervision, funding acquisition, and drafting and revising the article.

## Conflict of Interest Statement

The authors declare that the research was conducted in the absence of any commercial or financial relationships that could be construed as a potential conflict of interest.
